# The Prevalence and Surveillance of Surgical Site Infections in South Africa: A Literature Review

**DOI:** 10.1111/iwj.70690

**Published:** 2025-06-01

**Authors:** Emmy Ngoakoana Nokaneng, Samantha L. Holloway

**Affiliations:** ^1^ Maxillo‐Facial and Oral Surgeon University of Pretoria, Steve Biko Academic Hospital Complex Pretoria South Africa; ^2^ School of Medicine, Cardiff University Cardiff UK

**Keywords:** clinical indicator, oral and maxillofacial surgery, prevalence/incidence, surgical site infection, surveillance programme

## Abstract

Surgical site infection is a post‐operative complication, which has a significant clinical impact on the affected individual as well as the healthcare system. They are associated with poor outcomes such as increased length of hospital stay, morbidity, mortality and readmissions. As a result, surgical site infections are used as an indicator of the quality of surgical care and for benchmarking. The aim of the review is to gain insight on the current prevalence/incidence and surveillance of surgical site infection in South Africa. The objective was to determine the surgical site infection rate associated with Maxillo‐facial and Oral Surgery procedures. A literature review was conducted with the search strategy limited to articles published in English with no limitation to the period. Fifteen articles were deemed eligible for the review according to the inclusion criteria. Eleven articles focused on the epidemiology of surgical site infection in South Africa. The surgical site infection rate varies from 0.65‐48% with heterogeneity in the characteristics of the surveillance programmes. The review showed variability in the SSI rates with similar variability in the incidence of surgical site infection as reported on sub‐Saharan and African countries (7.93, 9.3, 19.1, 14.5% respectively). The above information was gleaned from institutional point/period prevalence or incidences due to a lack of an integrated national surveillance programme. Thus, there is an urgent necessity to establish an integrated national surveillance programme to facilitate monitoring as well as prevention of surgical site infection in South Africa.

**Summary**

- Surgical site infection (SSI) is a post‐operative complication, which has a significant clinical impact on the affected individual as well as the healthcare system. Thus, a healthcare system requires a surveillance programme to facilitate monitoring and preventative strategies.
- The aim of the review is to gain insight on the current prevalence/incidence and surveillance of SSI and to determine the SSI rate associated with Maxillo‐Facial and Oral surgery (MFOS) procedures in South Africa.
- A literature review from 1946 to march 2022 was conducted on three search engines.
- The findings show that South Africa does not have an established surveillance programme. To this end, the review suggested that the current indicator of SSI prevalence in SA is based on institutional point/period prevalence or incidences.
- It was shown that there is no evidence of reported SSI rates associated with MFOS in SA and thus a recommendation to conduct SSI surveillance associated with MFOS procedures.

## 1 Introduction

Surgical site infection (SSI) is a post‐operative complication, which has a significant clinical impact on the affected individual as well as the healthcare system [1]. SSIs are a common post‐operative complication, which are inextricably related to both clinical and financial outcomes of the patient and the health facility [1, 2]. SSIs are preventable; however, they are associated with poor outcomes such as increased length of hospital stay (LOS), morbidity, mortality and readmissions [1, 3, 4]. As a result, SSIs are used as an indicator of the quality of surgical care and for benchmarking although there is still no consensus on the quality of care indicators [5, 6, 7].

SSIs are associated with a negative impact on the post‐operative recovery, resulting in an increased LOS in patients diagnosed with an SSI in comparison with their counterparts [1, 8, 9]. In patients who had colorectal surgery, the median LOS was three times longer for patients with an SSI than the unaffected patients median 7.0 days (Interquartile range [IQR] 11.0 vs. 2.0 days; *p* < 0.001) [10]. Reviews on the socioeconomic impact of SSIs showed that the LOS varied between 2.8 and 54 days, whilst a further review showed delayed discharges in 34% of cases with an SSI [11, 12].

Similar findings were reported in South Africa (SA). Sonntag reported that 59% (*n* = 58/98) of the cohort with an SSI required re‐admission whilst 35.7% (*n* = 35/98) thereof were transferred to a higher level of care [13]. The mean LOS was 12.3 days (standard deviation [SD] ± 6.4 days, range 2–36 days). On the contrary, in a retrospective study at an academic tertiary hospital, the mean hospital stay associated with post‐caesarean section SSI was 5 days (range 2–32 days) [14].

Thakore et al. reported that patients with an SSI incurred greater costs than unaffected patients in all categories of services which were attributed to the initial hospitalisation as well as the readmission for the infection [15]. The costs for infected patients were $108 782 compared to $57 418 in the matched control group. According to Piednoir et al. [11] the total costs as a result of surgical revision contributed 37.9% of the total costs whereas 59.4% are borne by the need for hospitalisation [11]. Therefore, the measure of the financial impact of SSI assists in supporting the case for identifying interventions to reduce their occurrence [16].

The economic burden of SSI in SA remains poorly defined due to lack of an established national surveillance programme [17, 18]. It may be inferred from the published reports that the costs associated with SSI may be higher than in patients not affected by SSI with increased LOS, readmission, and procedures. Nair et al. reported that patients with healthcare‐associated infections (HAI) had a mean 20.8 days (95% CI, 12.1 to 29.4) LOS in comparison to 9.1 days (95% CI, 7.8 to 10.5) in unaffected patients [19]. Dell et al. [20] also reported a mean LOS of 9 days in comparison to 6 days in unaffected patients with additional procedures and re‐admission to intensive care unit (ICU) [20]. Similarly, the LOS associated with neurosurgical procedures in patients with SSI was more than 30 days (*p* = 0.008) [21].

The prevalence or incidence of SSI associated with Maxillo‐Facial and Oral Surgery (MFOS) procedures varies according to the type of the procedure with rates from 3.8% to 21.4% [12, 22, 23, 24]. The review of the literature suggests that it is lower in procedures such as orthognathic surgery and higher in head and neck cancer procedures [12, 25, 26]. Similarly, SSI within the speciality is associated with increased morbidity as well as financial burden on patients [12].

In SA, there is a dearth of information on the morbidity and mortality associated with SSI, partly due to the lack of a national surveillance programme [17, 27]. Thus, the aim of this literature review was to gain insight on the current prevalence/incidence of SSI in SA as well as to characterise SSI surveillance programmes. The review was part of a wider study to examine the data on the incidence of SSI within the Maxillo‐Facial and Oral Surgery speciality at the University of Pretoria/Steve Biko Academic Hospital Complex in the Gauteng Province. The surveillance study will be reported separately [28].

## 2 Methods

### 2.1 Search Strategy

The literature review entailed a review of cross‐sectional observational studies as well as the current standards in SSI prevention in SA which required the evaluation of audits/qualitative studies. Cooke et al. [29] suggested that the SPIDER tool is an appropriate framework to structure a search strategy for qualitative or mixed research [29]. The current project is a mixed method; it was considered appropriate to use the SPIDER tool for the search strategy.

The authors initially conducted an explorative exercise with the objective of “mapping rapidly” and to assess the extent of the literature and evidence available on SSI surveillance in SA [30]. The search strategy was conducted through MEDLINE, Embase, and Scopus. A supplementary search was conducted on Google scholar for grey literature. Due to limited publications within the South African context, the retrieved articles were cross‐referenced to identify additional and relevant published articles. The search strategy was limited to articles published in English with no limitation to the period MEDLINE (1946 to 25 March 2022); Embase (1947 to 25 March 2022; Scopus 2004 to 25 March 2022). Search strategies were conducted with a focus on the surveillance and epidemiology of SSI within the South African context.

### 2.2 Search Criteria

The search terms included (“surgical wound infection”[MeSH Terms] OR “surgical”[All Fields] AND “wound”[All Fields] AND “infection”[All Fields]) OR (“surgical wound infection”) [All Fields] OR (“surgical”[All Fields] AND “site”[All Fields] AND “infections”[All Fields]) OR (“surgical site infections”[All Fields] AND “surgery”[All Fields]) OR (“oral and maxillofacial surgery”[All Fields]).

### 2.3 The Inclusion and Exclusion Criteria

The inclusion criteria included articles that focused on the prevalence or incidence of SSI to determine current trends in prevalence/incidence of SSI rate and characteristics of the surveillance programmes. Furthermore, review articles on SSI surveillance in SA and guidelines on SSI surveillance were included to understand the current challenges with SSI surveillance as well as the SSI surveillance framework in SA. The review excluded studies with a focus on the prevalence or incidence of hospital acquired infections (HAI) without stratification of different types of infection and those not in English.

The inclusion and exclusion criteria for the review studies on the surveillance within the MFOS speciality included studies on the surveillance of SSI associated with MFOS procedures which included/described the SSI definition as well as those that indicated the wound classification utilised. The review excluded studies with a focus on the prevalence or incidence of HAI and those not in English.

### 2.4 Assessment of the Methodological Adequacy of the Evidence

The Joanna Briggs Institute (JBI) tool was utilised for the critical appraisal of methodology quality or risk of bias. As the JBI tool does not grade the overall strength of the evidence, the authors considered the number of positive attributes in relation to the eight domains assessed.

## 3 Results

### 3.1 The Search Results

**FIGURE 1:**
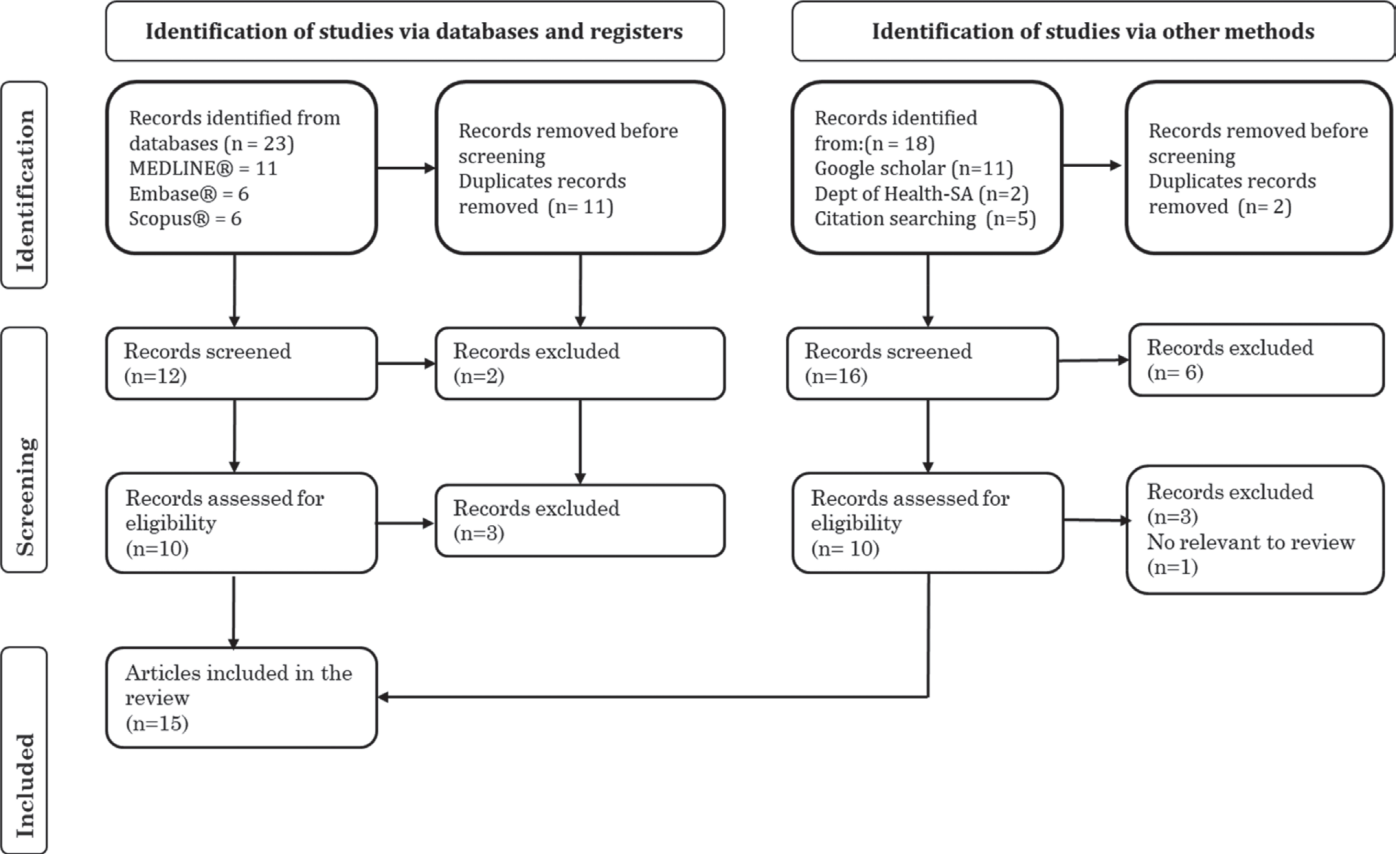
Flow chart for the literature review.

Forty‐one articles were identified through the search engines. Of these, 15 articles (including review articles and guidelines) were deemed eligible for the review according to the inclusion criteria (Figure 1).

Additionally, the inclusion considered the evolution of wound infection taxonomy (Table 1) as the use of SSI instead of “wound infection” was suggested and adopted in 1999 [37, 38]. The literature search did not identify any articles on surveillance in SA related to MFOS procedures (Table 2). Two review articles focused on the SSI surveillance systems/process and challenges in the South African context, whilst two were guidelines on SSI surveillance from the National Department of Health, SA (NDoH) [17, 45, 46, 47].

**TABLE 1:** Evolution of the taxonomy of surgical site infection.

Author	Definition
Berard and Gandon [31]	Wounds were considered uninfected if they healed per primam without discharge Definitely infected if there was a purulent discharge, whether or not organisms could be cultured from the purulent material Wounds that were inflamed without discharge and wounds that drained culture‐positive serous fluid were considered possibly infected Stitch abscesses were excluded from definite or possible infections
Pollock [32]	Wound sepsis is the discharge of pus. It is subdivided into: Primary (when the first discharge is pus) and Secondary (when the first discharge is not pus, but the discharging wound becomes colonised by bacteria from endogenous or exogenous sources). Both primary and secondary sepsis can be classified as minor (when constitutional disturbances are absent) and major (which makes the patient ill)
Polk et al. [33]	Wound infection has been defined as the emergence of pus from a wound, irrespective of the results of subsequent cultures. Indeed, any incision that must be opened for local care probably should be considered infected.
Garner et al. [34]	Surgical wound infection includes incisional surgical wound infection and deep surgical wound infection. Incisional surgical wound infection must meet the following criteria: Infection occurs at incision site within 30 days after surgery AND involves skin, subcutaneous tissue, or muscle located above the fascial. Deep surgical wound infection must meet the following criterion: Infection occurs at operative site within 30 days after surgery if no implant is left in place or within one year if implant is in place AND infection appears related to surgery, AND infection involves tissues or spaces at or beneath fascial layer
Consensus paper on the surveillance of surgical wound infections (1992), [35, 36]	The previous definitions of incisional surgical wound infection and deep soft tissue surgical wound infection’ are replaced by superficial incisional SSI and deep incisional SSI. Infections that involve the organ/space component of the surgical site were previously called deep surgical wound infections at specific sites other than soft tissue. These are now termed organ/space SSI and use the same specific sites as soft tissues. We introduce the term “organ/space” to define any part of the anatomy (e.g., organs or spaces), other than the incision, opened or manipulated during the operative procedure.

*Note:* *Adapted from Mehtar et al. with permission [37].

**TABLE 2:** Studies on the epidemiology of surgical site infection in South Africa.

Author(s)	Aim	Methodology	Prevalence type	Surveillance type	SSI^a^ definition	SWC^b^	Incidence/Prevalence (%)	MFOS^c^ rate
Sonntag [13]	To review deep incisional and organ/space SSI following CS at Mowbray Maternity Hospital, Western Cape	Retrospective hospital records	Period December 2011 to December 2014	Retrospective in‐patient medical records	Yes CDC^d^ (Horan et al. [35]) Focused only on deep incisional and organ/space SSI	No	*n* = 14 982 SSI = 0.65% (*n* = 98/14982) Y1^e^ = 0.88% SSI Y2^f^ = 0.90% SSI Y3^g^ = 0.70% SSI Deep incisional SSI = 75.5% (*n* = 74) Organ/space SSI = 24.5% (*n* = 24)	
Coetzer [14]	To audit post‐CS sepsis at Tygerberg Hospital, Western Cape. To determine the incidence of post CS^h^ sepsis.	Retrospective chart review	Incidence 1 February 2014 and 30 April 2014	In‐patient medical records	Yes CDC (Mangram et al. [36]) Focused only on superficial & deep SSI	No	*n* = 811 SSI rate = 3.82% (*n* = 27/811) Both superficial & deep incisional SSI	
Nair et al. [19]	To determine the point prevalence of healthcare associated infections at a tertiary hospital in Kimberley, Northern Cape	Prospective cross‐sectional	Point prevalence February 2016 to March 2016	Prospective active in‐patient	Yes CDC criteria (Emori et al. 1991)	No	*n* = 326 HAI^i^ = 7.67% (*n* = 25/326) SSI rate = 4.60% Superficial SSI = 38.5% (*n* = 10/15) Deep incisional SSI = 3.8% (*n* = 4/15)	No
Dell et al. [20]	To determine the incidence of HAI in trauma surgical wards To identify risk factors amenable to modification with a resultant reduction in infection rates.	Prospective descriptive analysis Groote Schuur Hospital, Cape Town	Incidence January to April 2018	Prospective active in‐patient	Not reported	No	*n* = 769 HAI = 0.03% (*n* = 22/769) SSI *n* = 12 Superficial *n* = 5 deep	No
Bokop‐Fotso et al. [21]	To determine the aetiology and incidence of hospital‐acquired infections and their associated risk factors following neurosurgical procedures.	Retrospective cross‐sectional Nelson Mandela Academic Hospital, Mthatha	Incidence October 2013 to September 2014	In‐patient Medical records Microbiology records	Not reported	No	*n* = 127 7.5% per year HAI (clinical) 4.2% per year HAI (microbiology) SSI = 48% (*n* = 61/127)	No
Bagratee et al. [39]	To determine whether prophylactic antibiotic administration using cefoxitin at the time of elective caesarean section significantly reduces infectious morbidity.	Prospective, double‐blind randomised placebo‐controlled trial King Edward VIII Hospital, Durban	Study period not reported	PDS^j^ at 6 weeks or prior pending on presenting symptoms (patient‐self assessment) Microbiology	Yes ‐Oral temp^k^ of ≥ 38°C on two occasions 6 h apart ‐Presence of wound cellulitis, erythema serous, serosanguinous and/or purulent discharge, with or without fever	No	Wound infection: *n* = 480 Placebo: 13.3% (*n* = 32/240) Cefoxitin: 12.5% (*n* = 30/240)	
Mulaudzi et al. [40]	To assess the influence of diabetes mellitus on early morbidity and mortality following a femoro‐popliteal bypass	Retrospective cross‐sectional Durban Metropolitan Vascular Service	Period 2001–2005	Retrospective record based	No Complications occurring within 30‐days of operation	No	*n* = 217 Wound infection: 5.8% (*n* = 6/102) of the DM^l^ 9.6% (*n* = 11/115) of the NDM^m^	
Johnson and Buchmann [41]	To determine the incidence of puerperal sepsis after CS in a group of South African women	Longitudinal descriptive Chris Hani Baragwanath Academic Hospital, Johannesburg.	Period 1 July to 13 August 2010	In‐patient PDS (telephonic interview 14 days after delivery)	Yes Author's criteria “Possible mild wound infection” ‐Temp ≥ 38°C ‐abnormal malodorous VG^n^ ‐abdominal pain or bleeding	No	*n* = 272 Wound infection: 12.5% (*n* = 34/272)	
Lebina et al. [42]	To determine the efficacy of routine prophylactic antibiotic use in the prevention wound‐related infections after circumcision	Retrospective cross‐observational	Incidence February 2011 and September 2011	Retrospective medical records review	Yes Author's criteria: symptoms or signs of wound related infection (crusting, inflammation/pus) mild/moderate/severe	No	*n* = 1291 Wound infection: 1% (11/1000)	
Brink et al. [43]	To implement improvement model for peri‐operative antibiotic prophylaxis utilising existing resources a survey of baseline SSI and compliance rates	Prospective audit 34 private hospitals in seven of nine South African provinces operated by Netcare Ltd.	Prevalence 1March 2013 and 1 September 2015	In‐patient medical records	Yes CDC (Horan et al. [35]) Focused only on superficial incisional or deep incisional SSI PDS	Yes	Composite SSI rate monthly Pre‐implementation SSI rate = 2.46% (95% CI 2.18–2.73) (12 months' period) Post‐implementation SSI rate = 1.97% (95% CI 1.79–2.15)	
Snyders et al. [44]	Analysis of the 30‐day readmission rate and underlying risk factors responsible for 30‐day readmission of general surgery patients	Retrospective Worcester Hospital, Western Cape Province	Period January 2014 to December 2017	Medical records PDS‐ readmission rate	No	Yes	SSI admission rate: 60.37% (*n* = 163/270) Clean/clean contaminated wounds: 82/202 (40.6) Dirty wounds: 120/202 (59.4)	

*Note:* ^a^SSI, surgical site infection; ^b^SWC, Surgical wound class; ^c^MFOS, Maxillo‐Facial and Oral surgery; ^d^CDC, Centres for Disease Control and Prevention; ^e^Y1, year one; ^f^Y2, year two; ^g^Y3, year three; ^h^CS, caesarean section; ^i^HAI, hospital acquired infections; ^j^PDS, post discharge surveillance; ^k^temp, temperature; ^l^DM, diabetes mellitus; ^m^NDM, non‐diabetes mellitus; ^n^VG, vaginal discharge.

#### 3.1.1 Excluded Publications

Seven articles were not eligible for the final review (electronic supplementary material).

#### 3.1.2 Assessment of the Methodological Adequacy of the Evidence

One publication was found to have achieved high methodological quality as the study achieved a high grade, suggesting a low risk of bias [19]. This was followed by four studies that achieved a moderate grading [13, 14, 43, 44]. Whilst six studies achieved a low grade, suggesting a high risk of bias [20, 39, 40, 41, 42]. Therefore, the poor grading achieved by the majority of the studies reviewed suggests that the studies have limited internal validity. This is consistent with the findings in the literature that observational studies have limited internal validity due to methodological heterogeneity, bias, and confounding factors [48]. In this review, most of the studies performed poorly in the domain of confounding factors, with a lack of identification of and strategies to deal with confounding factors [49].

## 4 Overview of the Findings of the Literature Review

The findings of the literature review will be discussed according to several themes including surveillance methods, study methodology (prevalence/incidence), SSI definition used, and surgical wound classification.

### 4.1 Surgical Site Infection Rates in South Africa

The reported SSI rate varies from 0.65% to 48% [13, 14, 19, 21, 39, 40, 41, 42].

Whilst some research has been carried out on the epidemiology of SSI within the South African context, a search of the literature revealed that no single study exists which explores SSI within the context of the MFOS speciality. As a result, there are no MFOS SSI rates which can be compared or contrasted with the global standards.

### 4.2 Characteristics of Surveillance Programmes

#### 4.2.1 Surveillance Methods

Six of the 11 studies used passive surveillance with reliance on in‐patient medical records for the identification of an SSI [13, 14, 21, 40, 42, 44]. Five studies utilised active prospective surveillance [19, 20, 39, 41, 43]. It is also interesting that only four studies in this review used a combination of methods for surveillance, that is, medical and microbiology records as well as post‐discharge surveillance (PDS) [19, 21, 40, 44].

Surveillance designs may be continuous or targeted to specific procedures or periods [7]. Similarly, the prevalence of SSI in SA is based on periods and points [13, 14, 19, 40, 41, 43, 44]. In relation to the procedures, surveillance designs targeted the following procedures: orthopaedic surgery, urology, trauma surgery, neurosurgery, general surgery, vascular surgery, thoracic surgery, breast oncology, obstetrics and gynaecology, and ophthalmology (Figure 3) [19, 20, 21, 39, 40, 41, 43].

Although Brink et al. and Nair et al. targeted various procedures, the authors did not identify any SSI associated with MFOS procedures [19, 43]. Similarly, Nair et al. reported that the orthopaedic ward contributed 15.56% (*n* = 7/25, CI 069–7.00) to the total HAI, suggesting a higher prevalence of SSI associated with orthopaedic procedures [19]. However, the authors did not report on SSI related to MFOS procedures.

#### 4.2.2 Post‐Discharge Surveillance

Four studies used post‐discharge surveillance (PDS) to monitor SSI [14, 39, 41, 44]. However, there was variation in the period of PDS, that is, 6 weeks, 14 days, and 30 days respectively [14, 39, 41, 44]. Both studies by Coetzer (2017) and Snyders et al. were aligned with the recommendation for the surveillance periods of 30 days or 90 days (with implants) which was a fair reflection of the SSI rate as the majority of patients with an SSI present with the infection post‐discharge from hospital [11, 35, 36, 50, 51, 52, 53].

### 4.3 Criteria for Defining Surgical Site Infection

In the context of the epidemiology of SSI in SA, there is variability in the definitions for SSI utilised. Four studies utilised the Centre for Disease Control and Prevention (CDC) criteria for SSI whereas three studies utilised their own criteria [13, 14, 19, 39, 41, 42, 44]. The remaining studies did not report the criteria used for the identification of an SSI [20, 21, 40, 44]. Although Bagratee et al. did not cite the use of CDC's definition, their criteria were aligned with that of CDC in terms of the clinical signs and symptoms, albeit the participants were surveyed for 6 weeks [35, 36, 39]. Similarly, Mulaudzi et al. monitored the participants for 30 days but did not report on the clinical criteria for the identification of the SSI [41].

The discrepancy in the SSI rate may be attributed to the type of SSI assessed. For example, Sonntag focused on deep incisional and organ/space SSI whereas Coetzer and Nair et al. focused on superficial incisional and deep incisional SSI [13, 14, 19]. Similarly, Brink et al. assessed the composite monthly SSI rate for both superficial incisional and deep incisional SSI [43].

### 4.4 Surgical Wound Classification

Two studies took into consideration the degree of wound contamination to assess the SSI rate [43, 44]. Synders et al. evaluated the wounds according to the four categories of surgical wound classification, whilst Brink et al. assessed clean and clean‐contaminated wounds [43, 44]. The remaining studies did not assess the degree of wound contamination.

## 5 Discussion

**FIGURE 2:**
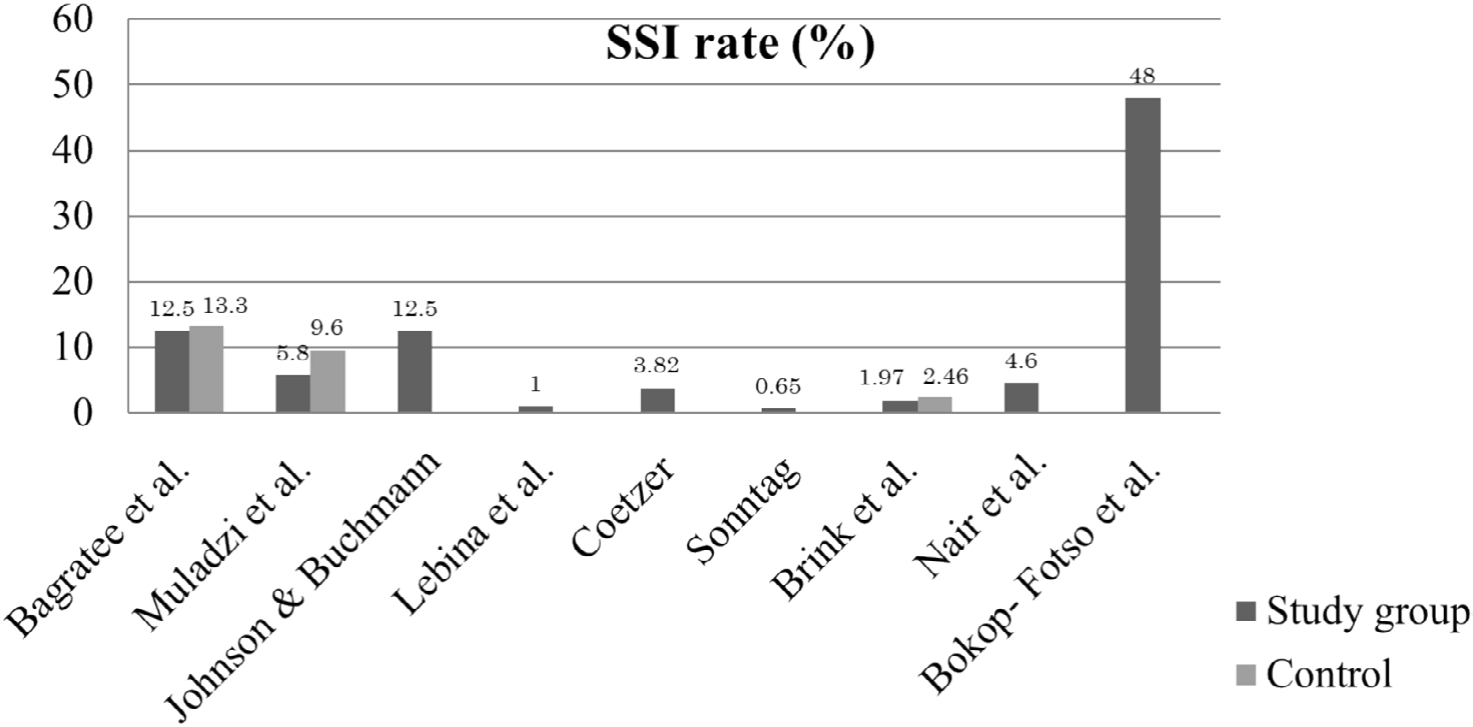
Incidence of the reported SSI rates in South Africa.

The literature review showed variability in the SSI rates (0.65%–48%) with similar variability in the incidence of SSI as reported in sub‐Saharan and African countries (7.93%, 9.3%, 19.1%,14.5%, respectively) (Figure 2) [54, 55, 56, 57]. However, these results should be interpreted with caution as comparisons remain valid if there are standardised surgical site infection surveillance programmes (SSISP) [3, 58, 59]. Although the rates are considerably higher than those in high‐income countries [10, 60]. Nonetheless, the reports provide a global view of the epidemiology of SSI in SA in contrast to other African countries. Whilst some research has been carried out on the epidemiology of SSI within the South African context, a search of the literature revealed that no single study exists which explored SSI within the context of the MFOS speciality.

**FIGURE 3:**
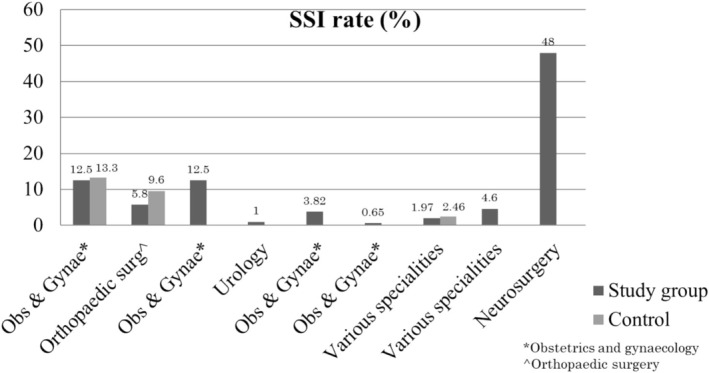
Incidence of the reported SSI rates in South Africa per speciality.

The above information was gleaned from institutional point/period prevalence or incidences due to a lack of an integrated national surveillance programme [18]. The data reviewed was derived from various surveillance programmes with both passive and active surveillance as well as a combination of surveillance methods. About 50% of the studies in this review used passive surveillance [13, 14, 21, 40, 42, 44] whilst the remaining studies utilised active surveillance [19, 20, 39, 41, 43]. Although the Centre for Disease Control—National Health Safety Network recommends that a combination of methods with the capacity to identify SSI may be utilised, prospective active, patient‐based surveillance has been and is still considered best practice as it identifies cases as they emerge [36, 51, 61, 62]. This suggests that the data collected by those studies with active prospective surveillance was reliable [62]. To the contrary, several authors have argued that the dependence on in‐patient surveillance models may not provide the true reflection of SSI rates [36, 63].

Less than 40% of the studies applied a combination of surveillance methods [19, 21, 39, 44]. A combination of methods may enhance the sensitivity of case identification [51]. On the other hand, Johnson and Buchman used patient self‐assessment by telephone for the identification of an SSI, albeit patient self‐assessment introduces confounding factors due to lack of training in wound assessment [51, 63, 64]. For example, confusing serous discharge with pus may result in under‐ and over‐reporting [63].

As some of the studies were cross‐sectional and retrospective in design [13, 14, 21, 40, 42], the identification of the infection in the cohort was solely record‐based without the clinical confirmation/diagnosis thereof by a surgeon/clinician as per CDC recommendation [13, 14, 21, 40, 42]. There was an assumption that the diagnosis by a treating clinician was aware of and familiar with the SSI case definition as per CDC definition [13, 14, 21, 40, 42]. Therefore, there was potential for misdiagnosis. Also, chart review is an unreliable and insensitive method for detecting an SSI as poor documentation may hinder case finding thus influence the reliability of the data [62, 65]. On the other hand, Aiken et al. suggested that a combination of a chart review and the daily inspection of the wound were not considered specific and sensitive methods [54].

Prospective active, patient‐based surveillance has been and is still considered best practice as it identifies cases as they emerge [51, 52, 61, 62]. Active surveillance has high sensitivity and specificity; however, it is a resource‐ and time‐consuming activity and therefore may not be feasible in settings with limited resources such as in SA [17, 27, 66].

Mahomed et al. identified various key challenges encountered with the implementation of HAI surveillance programmes in intensive care units within the South African healthcare system [17]. Foremost was the lack of organisational culture that promotes and enhances the implementation of surveillance programmes and data collection. Secondly, there was inadequate human resources, including a lack of personnel dedicated to data collection. As a result, the nurses felt overburdened by the process, whilst the infection control practitioners, as well as nursing managers, were unable to provide adequate supervision over the surveillance programmes due to conflicting work responsibilities. The implication of this is that missing data was a significant problem which resulted in the underestimation of the burden of disease. Additionally, the collected data was of poor quality, thus precluding the determination of the incidence or prevalence of HAI. This may be as a result of the lack of standardisation of diagnosis of HAIs, with diagnosis left to clinicians with no reference to standard criteria as recommended by CDC‐NHSN, which invariably led to poor quality data [67].

The majority of the studies under review lacked PDS (64%) [13, 19, 20, 21, 40, 42]. It has been suggested that the dependence on in‐patient surveillance models may not provide the true reflection of SSI rates as the patient may not necessarily report to the initial health facility [16, 36, 37, 68]. Additionally, with the evolving funding models, there is an expectation for a shorter hospital stay, thus making PDS a useful tool for the identification of SSI post‐discharge [68].

### 5.1 Surgical Site Infection Definition

The recognition of an SSI is dependent on the SSI case definition and consistent application thereof as this directly impacts the credibility of the data collected [59, 69]. Therefore, definitions utilised should have the ability to identify wound infection [70]. About 60% of the studies omitted to define the SSI or utilised their own criteria [39, 41, 42]. The finding is comparable to the global practices, that is, the inconsistencies in the application of SSI definitions [58, 59, 64]. These findings are consistent with other observations that showed that inconsistencies in the case definitions have partially influenced the disparities in the reported SSI rates [70]. For example, a systematic review on the quality of measurement of surgical wound infection as the basis for monitoring identified 41 SSI definitions in the literature [58].

Similarly, Leaper et al. reported that surgical site infection surveillance programme (SSISP) utilises case definitions based on the clinical presence of a discharge or inflammation, whereas some SSISP use CDC's definition which requires a diagnosis by the surgeon [59]. Leaper et al. observed considerable differences in the application of the CDC's definition within European institutions, with various “modifications” [64]. Notwithstanding that over the years, several amendments were made to the CDC's definition [36, 37, 50].

It is interesting to note that a significant proportion of SSIs reported by Nair et al. (*n* = 10/15, 38.5%) were from in‐patients with superficial incisional SSI [19]. Yet Kaye et al. were of the view that superficial incisional SSI was clinically insignificant in comparison to deep/organ space SSI due to the severity of deep/organ space SSI [71]. However, Lawson et al. showed that deep/organ space and superficial incisional SSI are different disease processes with different associated risk factors and, as such, should be surveyed and reported independently [72].

### 5.2 Surgical Wound Classification

The majority of the studies (80%) did not assess the degree of wound contamination and thus have limited internal validity as wound categories are regarded as indicators for the risk of an SSI [51, 73]. Accordingly, the risk of an SSI should be assessed with consideration to the degree of wound contamination whilst enabling comparisons to be drawn between the types of surgery [67, 74]. Evidence suggests that the risk of developing an SSI is directly correlated to the degree of wound contamination. For example, colorectal or gastrointestinal surgeries have a higher propensity for SSI as a result of a high burden of microbes within these sites [8, 75, 76]. Similarly, oncology‐related and transplant procedures are associated with a high incidence of SSI [77]. GlobalSurg Collaborative found that the highest incidence of SSI in a cohort of patients who had elective or emergency gastrointestinal surgery was in the dirty wounds group with 17.8% (*n* = 102/574) from high‐income, 31.4% (*n* = 74/236) from middle‐income, and 39.8% (*n* = 72/181) from low‐income countries [10].

## 6 Conclusion

A literature review was conducted to gain insight into the current evidence related to the SSI in SA based on the epidemiology of SSI, surveillance, and standards in SSI prevention. Furthermore, the epidemiology of SSIs in SA was contrasted to those in sub‐Saharan Africa and globally. According to Drawoski et al., the lack of an established active surveillance programme in SA has impeded the progress towards national or institutional SSI surveillance [18]. To this end, the review suggested that the current indicator of SSI prevalence in SA is based on institutional point/period prevalence or incidences with a lack of an integrated national surveillance programme.

The incidence of reported SSI rates in SA varies from 0.65% to 48% with similar variability in the incidence of SSI as reported in sub‐Saharan and African countries (7.93%, 9.3%, 19.1%, 14.5%, respectively) [13, 14, 19, 21, 39, 40, 41, 42, 43, 54, 55, 56, 57].

Taken together, the evidence suggests that there is variability in reported prevalence rates globally as well as locally. Fundamental to the variability is the inconsistent application of the SSI definitions and surgical wound classifications. The variability may also be attributed to the study methodologies, patient cohorts, and sample size [24].

The third objective of the review was to appraise the epidemiology of SSI in relation to the MFOS speciality. It was shown that there is no evidence of reported SSI rates associated with MFOS in SA and thus a recommendation to conduct SSI surveillance associated with MFOS procedures. In conclusion, there is an urgent necessity to establish an integrated national surveillance programme to facilitate monitoring as well as prevention of SSI in SA. The review informed the surveillance study that was undertaken in the Maxillo‐Facial and Oral Surgery unit at the University of Pretoria/Steve Biko Academic Hospital Complex and this is reported in a subsequent article [28].

## Conflicts of Interest

The authors declare no conflicts of interest.

## Data Availability

Data sharing is not applicable to this article as no new data were created or analyzed in this study.
